# Comparisons of Papanicolaou Utilization and Cervical Cancer Detection between Rural and Urban Women in Taiwan

**DOI:** 10.3390/ijerph18010149

**Published:** 2020-12-28

**Authors:** Chiu-Ming Yang, Fung-Chang Sung, Chao-Song Hsue, Chih-Hsin Muo, Shu-Wei Wang, Shwn-Huey Shieh

**Affiliations:** 1 Department of Health Services Administration, College of Public Health, China Medical University, Taichung 404, Taiwan; sanann861109@gmail.com (C.-M.Y.); fsung1008@yahoo.com (F.-C.S.); 2 Management Office for Health Data, China Medical University Hospital, Taichung 404, Taiwan; b8507006@gmail.com; 3 Department of Food Nutrition and Health Biotechnology, Asia University, Taichung 413, Taiwan; 4 China Medical University Bei Kang Hospital, Bei Kang, Yunlin County 651, Taiwan; 22057628@yahoo.com.tw (C.-S.H.); bage10312@gmail.com (S.-W.W.); 5 Feng Yuan Hospital, Ministry of Health and Welfare, Taichung 420, Taiwan; 6 School of Nursing, Kaohsiung Medical University, Kaohsiung 807, Taiwan; 7 Department of Nursing, Asia University, Taichung 41354, Taiwan

**Keywords:** cervical cancer, insurance claims data, Papanicolaou test, rural, urban

## Abstract

Using the claims data of one million insured residents in Taiwan from 1996–2013, this study identified 12,126 women in an urban city (Taichung) and 7229 women in a rural county (Yunlin), aged 20 and above. We compared Papanicolaou (Pap) test uses and cervical cancer detection rates between urban and rural women. Results showed that the Pap screening rate was slightly higher in rural women than in urban women (86.1 vs. 81.3 percent). The cervical cancer incidence was much greater for women without Pap test than women with the test (35.8 vs. 9.00 per 1000 in rural women and 20.3 vs. 7.00 per 1000 in urban women). Nested case-control analysis showed that Pap test receivers had an adjusted odds ratio (OR) of 0.35 (95% CI = 0.25–0.51) to be diagnosed with cervical cancer as compared to those who did not receive the test. The rural women had an adjusted OR of 1.46 (95% CI = 1.03–2.06) to be diagnosed with cervical cancer as compared to urban women. In conclusion, women in rural area are at higher cancer risk than city women. Women who do not undergo Pap tests deserve timely intervention of Pap test to prevent the onset of cancer, particularly in rural women with low income.

## 1. Introduction

Cervical cancer is a commonly detected cancer worldwide, and currently, more than a half million women are diagnosed with the disease each year [1,2]. Most patients with this cancer are women living in areas with low and middle income. The 5-year survival rate for the cancer has increased dramatically worldwide, particularly in developed countries, with an overall survival rate of over 65% in European countries and in the U.S. [3,4]. It is largely perceived that women in developed countries are more likely to have proper implementation of screening programs, which promotes early diagnosis and treatment and thus reduces the occurrence of cervical cancer and increases the survival rate of the patients [2,3,5,6]. Early detection and prompt treatment can prevent the progression of the disease, which can improve the survival rate. Approximately 90% of women who die from this cancer are women in countries with poor access to prevention and screening programs or treatments for precancerous conditions [7].

The Papanicolaou (Pap) test, liquid-based cytology, the HPV DNA testing and the visual inspection with acetic acid are some of the methods for screening the disease [8,9]. Among them, the Pap test is the most commonly used method to screen precancerous and cancerous processes in the cervix. It has also been deemed as a highly cost-effective public health intervention that has played an important role in mass population screening for detecting cancer in Asian women [10,11]. Culture has long been recognized as one of the most powerful factors influencing health beliefs and health-related behaviors [12,13]. Screening practices also vary among Asian areas, while Asian women share some similar concerns, such as access to screening, communication, and embarrassment.

The Pap test has also been adapted in Taiwan as a critical strategy to screen for cervical dysplasia. Gynecological clinics offered free Pap tests in the years 1974–1984 [14,15]. However, the use of this service was very low, particularly in women with disabilities [14,15,16,17,18]. During this period, it was observed that less than 5% of eligible women in Taiwan used the test [19]. According to the Ministry of Health and Welfare in Taiwan, National Health Insurance in Taiwan started providing free Pap test services for all eligible women when the insurance program was launched in 1995. The Formosa Cancer Foundation has also operated mobile cervical cancer screening clinics since 2000 to provide the test free, mainly targeting women living in rural villages in order to increase the screening rate in the hard to reach areas. The Ministry of Health and Welfare further sponsored hospitals to conduct outreach services and used mobile van screening clinics to promote Pap testing for underserved women in remote areas. Bilingual health workers were available to send out letters to notify residents in townships and villages about the dates, times, and locations of the screening services. The screening rate has thus increased annually. Studies found that 56.2% of general female adults have received Pap tests from 2004 to 2006, and 57.7% of female medical professionals have received tests from 2008 to 2010 [20,21]. The health promotion and disease prevention efforts have improved the screening rate, enabling 82.4% of eligible women to receive the test at least once within 3 years [22]. However, whether social disparities remain in screening compliance, as well as the associations of this with cervical cancer risk, deserve further study.

This study used Taiwan’s insurance claims data to evaluate whether screening behaviors are comparable between women living in an urban city (Taichung) and women living in a rural county (Yunlin) in central Taiwan. We also attempted to compare the cervical cancer risk between women with and without Pap tests in these two areas. Taichung is the second largest urbanized metro city in Taiwan. The population density in Taichung City was approximately 10-fold greater than that in Yunlin County, while the physicians/1000 people ratio was 3-fold greater. There were far more health care facilities in the city, and the average household income was also higher (Supplement Table S1).

## 2. Materials and Methods

### 2.1. Study Population

**Figure 1:**
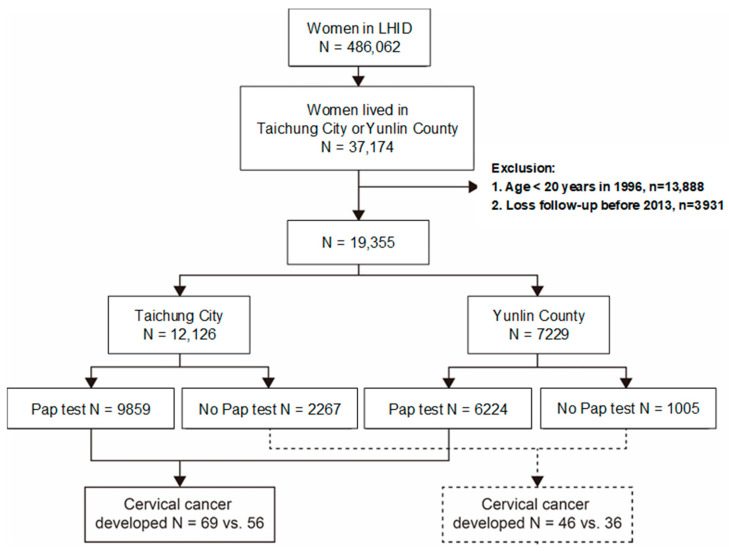
Flow chat for the process to identify the study population from the Longitudinal Health Insurance Database (LHID).

The National Health Insurance of Taiwan is a universal health insurance system, established in 1995, with 96% of 2.3 million people covered and over 95% of hospitals and clinics contracted by the end of 1996 [23]. This study obtained the Longitudinal Health Insurance Database (LHID) from the National Health Research Institutes (NHRI), which included the claims data of one million insured population randomly selected from a total of 23 million residents in Taiwan. From this database, we identified all women aged 20 years and older: 12,126 from the urban city of Taichung and 7229 from the rural county of Yunlin, which are both located in central Taiwan and covered in the insurance scheme of 1996–2013 (Figure 1).

From both the outpatient and inpatient claims data of these women, we identified those who had received the Pap smear test and those who had been diagnosed with cervical cancer in the years 1996–2013. For the identified population, the Pap smears received from 1996–2013 were measured, and the diagnoses of cervical cancer were identified from both outpatient and inpatient claims data. Pap smear claims were reported by the insurance policy with special codes, as a sequence of 31, 32 and 35, representing annual Pap test, every 3-year Pap test and pregnant clinic Pap test, respectively. The insurance system requires that Pap smears are collected in conjunction with a pelvic exam for women 30 years old and above and for sexually active women between 20–29 years. The smears are taken from the cervix by an obstetric-gynecologic doctor, a family doctor at health institutions, or by other trained and registered personnel at local public health departments or in mobile clinics. The cell samples are examined by a pathologist or by an expert trained and certificated by the Health Promotion Administration, and suspicious cells require further testing and diagnosis. If the Pap smear cells appear abnormal and the pathologist is certain a cancer is present, the patient can be certified in a catastrophic category after a second physician confirms the status. The cervical cancer diagnosis and claims were recorded using the International Classification of Diseases, Ninth Revision, and Clinical Modification with their codes being 179, 180, 184, 184.4 and 184.9 or A-code 120 and 122, respectively.

### 2.2. Statistical Analysis

We first compared the distributions of stratified groups with regard to the age, number of clinical visits annually, and income level of urban and rural women who had ever received the Pap smear examination and those who did not undergo the test. Incidence case and rate of cervical cancer being diagnosed were then calculated for each stratum. The incidence of women with Pap test to the incidence of women without Pap test relative risk (RR) and 95% confidence interval (CI) were calculated for all strata for the urban and rural women separately. We further conducted a nested case-control analysis by including all women who were detected with cervical cancer, therefore referred to as cases. The control group included a random selection of women who were free of cervical cancer, with the sample size 4-fold of cases. We used logistic regression analysis to calculate the odds ratio (OR) of cervical cancer and related 95% confidence interval (CI) associated with Pap screening status, as well as the urban and rural status. Multivariable analysis was used to calculate an adjusted odds ratio (aOR) after controlling for age and income. We further estimated the RR of cancer in rural to urban women with and without the Pap test by stratified age, frequency of clinic visits, and income.

## 3. Results

### 3.1. Baseline Characteristics of Study Population

The Pap screening rate among the 19,355 women included in this study was higher in the rural area than in the urban area (86.1 vs. 81.3%), with an OR of 1.50 (*p* = 0.0123) (Table 1). In this study, there were more women aged 20–49 years in the urban Taichung than in the rural Yunlin area (86.6% vs. 71.1%). The rural women had more clinical visits and higher income than the urban women. The portion of women with 20 or more frequent clinical visits and a Pap screening test was higher in Yunlin than in Taichung (47.3 vs. 41.7%).

### 3.2. Incidence of Cervical Cancer

Table 2 shows that the incidence of cervical cancer in the rural women was greater than in the urban women and was greater in women without the test than in those with the test; the gap was greater in the rural area (35.8 vs. 9.0%, or nearly 4 folds) than in the urban area (20.3 vs. 7.0%, or nearly 3 folds). In other words, the incidence rate was 1.76 folds greater for women without the test or 1.29 folds greater for women with the test in Yunlin County, as compared with the incidence rates in Taichung City. The Pap test users were at reduced risk of cervical cancer, with an adjusted RR of 0.38 (95% confidence interval = 0.25–0.56) for the urban women, and 0.25 (95% confidence interval = 0.17–0.39) for the rural women. The age-specific incidence among non-Pap test groups peaked in the age group of 50–64 years old in both Taichung City and Yunlin County (39.6 vs. 62.8 per 1000). The incidence rate increased as the number of clinical visits increased in women who did not perform the screening test but not in the women who had the screening test performed. Among the group of women with more than 30 visits, the incidence difference between women without the test and women with the test was greater in the rural county than in the urban city (119.0 vs. 65.6 per 1000), with the adjusted RRs of 0.07 and 0.10, respectively, for users. We also noted that the cancer incidence rate was higher in middle income women without screening, and it was higher in Yunlin County than in Taichung City (46.4 vs. 34.6 per 1000). At the same time, the cancer incidence rate was lower in middle income women who had received the screening test, and it was higher in Yunlin County than in Taichung City (7.24 vs. 5.37 per 1000).

### 3.3. Nested Case-Control Analysis

During the research period, we identified 207 cases of cervical cancer in the study population (Table 3). The Pap screening rate was lower in the cases than in the controls (60.4 vs. 80.9 %), with an aOR of 0.35 (95% CI = 0.25–0.51). Women with cervical cancer were older than the controls, and the aOR of cancer increased with age. While the risk of being diagnosed with cervical cancer was higher for those with higher clinic visits and higher income, the difference was not significant. Rural women were at a higher risk of being diagnosed with cervical cancer, with an aOR of 1.46 (95% CI = 1.03–2.06).

## 4. Discussion

For decades, health care authorities have confidently insisted on the effectiveness of Pap smear test in contributing to the early detection of cervical cancer so that timely and appropriate diagnosis and treatment can be provided [1,2,9]. Evidence has shown that widespread screening programs greatly reduce the cervical cancer incidence and deaths from the cancer. The screening programs also contribute to cancer detection in populations of various socioeconomic levels [9,24,25,26].

Disparities in cancer screening among populations are well documented worldwide, and the screening rates are higher in women in developed countries than women in developing countries. Disparities in screening have also been shown to exist among ethnic groups in the US. The number of Chinese women screened for cervical cancer within 3 years was lower than Filipina women, 68.7 vs. 82.7% [13]. However, the probability of being diagnosed with cervical cancer in the U.S. has been reduced to 1/162, as compared to 1/8 for breast cancer, as the majority of women had high compliance in cancer prevention [27]. On the other hand, cervical cancer remains a significant cause of mortality among Asian women, but the compliance rates of Pap tests are also much lower than those of Western countries [11,15,28,29,30]. In the U.S., the Medicaid-insured population has a lower rate of Pap test utilization [30].

An earlier Lithuanian survey found that the amount of women who had participated in the Nationwide Cervical Cancer Screening Program was lower in urban areas than in rural areas (9.6 vs. 14.7%) [31]; however, a reminder letter increased attendance to 24.6% and 30.8%, respectively. In the UK, the 5-year coverage of the Pap test rate in women ranged between 77.8%–79.0% in 2011–2012 [32]. Cancer has been the leading cause of death for decades in South Korea [33]. In order to reduce the cancer mortality rate, South Korea established the National Cancer Screening Program for the population to provide cancer screening for 5 major cancers, including cervical cancer using the Pap test, and the screening rate increased annually by 1.3% to 67.0% in 2013 [34]. This screening rate was confirmed by a focus group study [35].

Cervical cancer was once the most frequently diagnosed cancer among the women of Taiwan, accounting for 20% of all female cancers in 1960s [36]. The Pap smear screening test might be one of important factors to have contributed to reducing the disease by 66%, from 25 per 100,000 in 1995 to 8.5 per 100,000 in 2015 [22]. The disease recently became the 9th most commonly detected cancer in women of Taiwan. Our data show that for women with at least one lifetime screening of Pap test, the cervical cancer incidence can be reduced for 65% as compared to women who have never received the screening test. This finding is consistent with a recent finding seen in Uganda, where screening once in a lifetime could yield a sufficient reduction of cervical cancer up to 70% [37]. A population-based case-control study in England also found that regular cervical cancer screening could reduce 70% of deaths from this cancer [24].

Our findings reflect that the Health Insurance Program initiated Pap screening strategy seems to make a contribution to reducing the cancer risk, although there has been slow progress in compliance with the test. The progress could be advanced further if the screening service is increased.

In this present study, most insured women had received at least one Pap test during the study period. To our surprise, Pap test utilization was slightly higher for the rural women than for the urban women. Rural women might increase their access to the detection services with the availability of mobile screening units [28,38]. However, the incidence of cervical cancer diagnosis was higher in the rural women than in the urban women, with a gap greater in those without the Pap screening (35.8 vs. 20.3 per 1000) than those with the screening test (9.00 vs. 7.00 per 1000). The risk of being diagnosed with cervical cancer for women without the test, as compared to women with the test, was greater in the rural county than in the urban city. The rural-urban difference of cervical cancer incidences among women without the test was particularly large in those aged 50 years and older. It is important to promote screening tests for older rural women, as they are likely reluctant to utilize the screening services.

This study also showed that the rural women had more clinical visits than the urban women. The detection of cervical cancer in women without Pap screening test relied on frequent clinical visits. The incidence increased with the frequency of visits. For women with more than 30 clinical visits, the cervical cancer incidence rate was 1.8-fold greater in rural women than in urban women. Thus, detection inequality consistently existed among the rural and urban women, with and without the Pap test. The overall cervical cancer risk was 46% greater in the rural women than in the urban women. In general, education level is lower among rural women than in urban women in Taiwan, particularly for older women. We conducted a further data analysis to compare the relative risk of cancer in rural to urban women with and without Pap test by age, clinic visit, and income. Results show higher risks for rural women, but the risk was significant only in the low-income group without the Pap test (Supplement Table S2). Most were not significant, probably because of the small group size of cases in the stratified analysis.

The strength of this study was its use of large population-based insurance claims data to compare the extent of cervical cancer detection between rural and urban women with and without Pap smears, as the insurance claims data reflect the real-world practices of Pap testing and cancer detection in women. All cancer patients were diagnosed based on the pathological reports confirmed by specialists. Moreover, because costs for these services were reimbursed to the healthcare providers through the insurance system based on their claims, the data were reliable. Our findings demonstrate that the difference that exists between rural and urban women could be associated with the variations in their economic growth. However, detailed information regarding socioeconomic status was unavailable in the claims data. We were unable to observe whether the use of the Pap test and cancer detection were associated with women’s education level and therefore with knowledge about and attitude to cancer prevention. The other potential limitation in this study is that we compared the occurrence of cervical cancer between women with and without Pap testing instead of using the follow-up method to calculate the incidences and hazard ratio of developing cervical cancer. Estimations of cancer risks in the follow-up design could be different from the findings in this present study. In addition, information regarding cancer stages was not available in our claims data to assess the distribution of the disease by stage.

## 5. Conclusions

Over 80% of women who were included in this study had received the Pap smear screening test at least once during the study period, without disparity between rural and urban women. However, the cervical cancer incidence was greater in the rural women than in the urban women, even in women with the screening test. Nearly, 60% of cancer cases occurred in women who had received the test versus 81% controls who had the test. We observed that women who belonged to older age group or had frequent clinical visits were more likely to be diagnosed with the cancer. Further efforts should be made to improve women’s utilization of the screening test, particularly targeting older women who are at a higher risk and younger women who are reluctant to have the Pap test services.

## Figures and Tables

**Table 1:** Comparison between women who ever received and who never had Pap smear screening test by age, outpatient visits, and income among women in Taichung city and Yunlin County.

	Taichung City Pap Smear Test				Yunlin County Pap Smear Test				*p*-Value
	No N = 2267 (18.7%)		Yes N = 9859 (81.3%)		No N = 1005 (13.9%)		Yes N = 6224 (86.1%)		
Age in 1996	n	(%)	n	(%)	n	(%)	n	(%)	<0.0001
20–29 yrs	783	(34.5)	3518	(35.7)	276	(27.5)	1545	(24.8)	
30–49	920	(40.6)	5021	(50.9)	361	(35.9)	2882	(46.3)	
50–64	404	(17.8)	1128	(11.4)	239	(23.8)	1462	(23.5)	
≥65	160	(7.06)	192	(1.95)	129	(12.8)	335	(5.38)	
*p*-value	<0.0001				<0.0001				
Outpatient visits									<0.0001
0–9	1134	(50.0)	1980	(20.1)	470	(46.8)	1107	(17.8)	
10–19	573	(25.3)	3771	(38.3)	270	(26.9)	2174	(34.9)	
20–29	301	(13.3)	2190	(22.2)	139	(13.8)	1471	(23.6)	
30+	259	(11.4)	1918	(19.5)	126	(12.5)	1472	(23.7)	
*p*-value	<0.0001				<0.0001				
Income, NTD									<0.0001
<20,000	1723	(76.0)	7034	(71.4)	583	(58.0)	3403	(54.7)	
20,000–39,999	433	(19.1)	2048	(20.8)	388	(38.6)	2486	(39.9)	
≥40,000	111	(4.90)	777	(7.88)	34	(3.38)	335	(5.38)	
*p*-value	<0.0001				0.01				

Pap smear screening: card_seq_no = 31, 32, 35.

**Table 2:** Cervical cancer cases diagnosed and compared between every Pap smear screened and not screened by age, yearly outpatients visit, and income among women in Taichung city and Yunlin country, 1996–2013.

Taichung City	None Screening		Pap Screening		RR (95% CI)	
	Case	Rate	Case	Rate	Crude	Adjusted
	n	(per 1000)	n	(per 1000)		
Age in 1996						
20–29 years	3	(3.83)	7	(1.99)	0.52 (0.13–2.01)	0.59 (0.14–2.51)
30–49	21	(22.8)	45	(8.96)	0.39 (0.23–0.66)	0.35 (0.20–0.59)
50–64	16	(39.6)	14	(12.4)	0.31 (0.15–0.64)	0.28 (0.14–0.59)
≥65	6	(37.5)	3	(15.6)	0.42 (0.10–1.67)	0.35 (0.08–1.24)
Outpatient visits						
0–9	11	(9.70)	10	(5.05)	0.52 (0.22–1.23)	0.61 (0.25–1.47)
10–19	11	(19.2)	30	(7.96)	0.41 (0.21–0.83)	0.65 (0.31–1.36)
20–29	7	(23.3)	17	(7.76)	0.33 (0.14–0.80)	0.46 (0.17–1.20)
30+	17	(65.6)	12	(6.26)	0.10 (0.05–0.20)	0.10 (0.05–0.22)
Income						
<20,000	28	(16.3)	53	(7.53)	0.46 (0.29–0.73)	0.54 (0.34–0.88)
20,000–39,999	15	(34.6)	11	(5.37)	0.16 (0.07–0.34)	0.16 (0.07–0.35)
≥40,000	3	(27.0)	5	(6.44)	0.24 (0.06–0.99)	0.18 (0.04–0.79)
Total	46	(20.3)	69	(7.00)	0.34 (0.24–0.50)	0.38 (0.25–0.56)
**Yunlin County**						
Age in 1996						
20–29 years	2	(7.25)	6	(3.88)	0.54 (0.11–2.66)	0.49 (0.09–2.68)
30–49	14	(38.8)	31	(10.8)	0.28 (0.15–0.52)	0.27 (0.14–0.52)
50–64	15	(62.8)	12	(8.21)	0.13 (0.06–0.28)	0.12 (0.06–0.27)
≥65	5	(38.8)	7	(20.9)	0.54 (0.17–1.70)	0.55 (0.17–1.74)
Outpatient visits						
0–9	7	(14.9)	10	(9.03)	0.61 (0.23–1.59)	0.68 (0.25–1.81)
10–19	7	(25.9)	18	(8.28)	0.32 (0.13–0.76)	0.41 (0.17–0.99)
20–29	7	(50.4)	14	(9.52)	0.19 (0.08–0.47)	0.21 (0.08–0.54)
30+	15	(119.0)	14	(9.51)	0.08 (0.04–0.17)	0.07 (0.03–0.15)
Income						
<20,000	17	(29.2)	34	(9.99)	0.34 (0.19–0.61)	0.30 (0.16–0.54)
20,000–39,999	18	(46.4)	18	(7.24)	0.16 (0.08–0.30)	0.17 (0.09–0.32)
≥40,000	1	(29.4)	4	(11.9)	0.41 (0.05–3.63)	0.47 (0.05–4.75)
Total	36	(35.8)	56	(9.00)	0.25 (0.17–0.38)	0.25 (0.17–0.39)

RR, relative risk; CI, confidence interval. Adjusted RR, measured using multivariables including age, outpatient visits, and income.

**Table 3:** Nested case-control analysis for measuring factors associated with diagnosed cervical cancer in women in Taichung city and Yunlin county combined.

	Controls N = 828		Cases N = 207		Total N = 1035		Univariable		Multivariable	
	n	(%)	n	%	n	%	OR	(95% CI)	OR	(95% CI)
Pap screening										
No	158	(19.1)	82	(39.6)	240	(23.2)	1.00	(reference)	1.00	(reference)
Yes	570	(80.9)	125	(60.4)	729	(76.9)	0.42	(0.26–0.50)	0.35	(0.25–0.51)
Age in 1996										
20–29	281	(33.9)	18	(8.70)	299	(28.9)	1.00	(reference)	1.00	(reference)
30–49	395	(47.7)	111	(53.6)	506	(48.9)	4.39	(2.58–7.26)	3.93	(2.48–7.40)
50–64	120	(14.5)	57	(27.5)	177	(17.1)	7.42	(4.70–9.23)	6.45	(4.11–8.72)
≥65	32	(3.86)	21	(10.1)	53	(5.12)	10.2	(4.20–23.8)	7.01	(3.21–18.7)
Outpatient visits										
0–9	208	(25.1)	38	(18.4)	246	(23.8)	1.00	(reference)	1.00	(reference)
10–19	289	(34.9)	66	(31.9)	355	(34.3)	1.25	(0.81–1.94)	1.56	(0.96–2.52)
20–29	174	(21.0)	45	(21.7)	219	(21.2)	1.42	(0.88–2.28)	1.47	(0.87–2.50)
30+	157	(19.0)	58	(28.0)	215	(20.8)	2.02	(1.28–3.20)	1.42	(0.85–2.36)
Income										
<20,000	557	(67.3)	132	(63.8)	689	(66.6)	1.00	(reference)	1.00	(reference)
20,000–39,999	221	(26.7)	62	(30.0)	283	(27.3)	1.18	(0.84–1.66)	0.89	(0.61–1.30)
≥40,000	50	(6.04)	13	(6.28)	63	(6.09)	1.10	(0.58–2.08)	1.13	(0.58–2.22)
Area										
Taichung city	540	(65.2)	115	(55.6)	655	(63.3)	1.00	(reference)	1.00	(reference)
Yunlin County	288	(34.8)	92	(44.4)	380	(36.7)	1.50	(1.10–2.04)	1.46	(1.03–2.06)

## Data Availability

This study used data obtained from the National Health Research Institutes. The authors are not allowed to duplicate the raw data. Researchers may apply for data from the Ministry of Health and Welfare of Taiwan.

## Supplementary Materials

The following are available online at https://www.mdpi.com/1660-4601/18/1/149/s1, Table S1: Population density, physician/1000 people, number of medical center and other types of health care facility, hospital and income level of Taichung city and Yunlin county in 2000, Table S2: Yunlin women to Taichung women relative risk of cervical cancer for women with and without Pap test
