# Supplementary material for: Comparisons of Papanicolaou Utilization and Cervical Cancer Detection between Rural and Urban Women in Taiwan
Source: Int J Environ Res Public Health. 2020 Dec 28;18(1):149. doi: 10.3390/ijerph18010149 (PMC7795661; doi:10.3390/ijerph18010149)
Supplement: Supplementary file 1 [file ijerph-18-00149-s001.pdf]

Supplement Table S1. Population density, physician/1000 people, number of medical center and other types of health care facility, hospital and income level of Taichung city and Yunlin county in 2000

Variable	**Taichung city**	**Yunlin county**
Population density (people/ km^2)[1]	5909.7	575.9
Physician/1000 people[1,2]	12.7	4.03
Medical center	5	0
Other health facility	2028	815
Monthly income, NTD		
<20,000	67856 (84.8)	32637 (90.8)
20,000–39,999	9188 (11.5)	2463 (6.85)
≥40,000	2994 (3.74)	856 (2.38)

[1] Data source: https://www1.stat.gov.tw/ct.asp?xItem=15408&CtNode=4692&mp=3

[2] Data source: https://statdb.dgbas.gov.tw/pxweb/dialog/CityItemlist_o.asp#

Supplement Table S2. Yunlin women to Taichung women relative risk of cervical cancer for women with and without Pap test

	**Pap test**			
	**Yes**		**No**	
**Variable**	**Crude RR (95% CI)**	**Adjusted RR (95% CI)**	**Crude RR (95% CI)**	**Adjusted RR (95% CI)**
Age in 1996				
20–29 yrs	1.95 (0.66-5.81)	1.91 (0.64-5.67)	1.89 (0.32-11.3)	1.96 (0.33-11.7)
30–49	1.20 (0.76-1.90)	1.27 (0.80-2.01)	1.70 (0.86-3.34)	1.27 (0.62-2.63)
50–64	0.66 (0.31-1.43)	0.66 (0.27-1.60)	1.58 (0.78-3.21)	1.62 (0.72-3.62)
≥65	1.34 (0.35-5.17)	2.06 (0.45-9.34)	1.03 (0.32-3.39)	2.07 (0.53-8.09)
Outpatient visits				
0–9	1.79 (0.74-4.30)	1.20 (0.46-3.12)	1.54 (0.60-3.96)	1.00 (0.36-2.79)
10–19	1.04 (0.58-1.87)	0.93 (0.51-1.72)	1.35 (0.52-3.48)	1.00 (0.35-2.81)
20–29	1.23 (0.60-2.49)	1.12 (0.53-2.38)	2.17 (0.76-6.17)	1.93 (0.62-5.94)
30+	1.52 (0.70-3.29)	1.82 (0.82-4.03)	1.81 (0.91-3.63)	1.73 (0.83-3.61)
Income				
<20,000	1.33 (0.86-2.04)	1.26 (0.82-1.94)	1.79 (1.08-3.28)	2.15 (1.16-3.96)
20,000–39,999	1.35 (0.64-2.85)	0.78 (0.33-1.86)	1.34 (0.67-2.66)	1.02 (0.47-2.23)
≥40,000	1.86 (0.50-6.91)	1.76 (0.47-6.56)	1.09 (0.11-10.5)	2.50 (0.19-32.3)

RR, relative risk; CI, confidence interval; Adjusted RR, measured with multivariables including age, outpatient visits, and income.
